# Primary Splenic Angiosarcoma Presenting as Idiopathic Thrombocytopenic Purpura: A Case Report and Review of the Literature

**DOI:** 10.1155/2016/4173060

**Published:** 2016-08-29

**Authors:** S. Christopher N. Frontario, Anna Goldenberg-Sandau, Darshan Roy, Roy Sandau

**Affiliations:** ^1^Rowan University School of Osteopathic Medicine, Kennedy University Hospital, Department of Surgery, 42 East Laurel Road, Suite 2500, Stratford, NJ 08084, USA; ^2^Rowan University School of Osteopathic Medicine, Kennedy University Hospital, Department of Pathology, 42 East Laurel Road, Suite 2600, Stratford, NJ 08084, USA

## Abstract

Angiosarcoma of the spleen is a rare malignancy that arises from vascular endothelial origin. This neoplasm is highly malignant and diagnosis is often delayed due to the vague presentation of clinical symptoms. A case report and concise review of the current diagnostic criteria and surgical treatment are provided to aid in the detection and treatment of this malignancy. We present a case of a 56-year-old female who presented with massive splenomegaly secondary to angiosarcoma of the spleen. The patient suffered from longstanding symptomatic anemia and thrombocytopenia. Diagnosis of a splenic angiosarcoma can be difficult due to the vague presentation and lack of concrete risk factors. Early identification and splenectomy are paramount. However, it is an aggressive malignancy with poor prognosis. We reviewed the literature of the current diagnostic and surgical treatment of primary splenic angiosarcoma.

## 1. Introduction

Primary malignancies of the spleen are categorized into vascular and lymphoid with the former originating from red pulp and the latter from white pulp [1]. Angiosarcoma of the spleen is a rare and aggressive neoplasm that arises from vascular endothelium. The diagnosis and treatment of primary splenic angiosarcoma are commonly delayed due to the varied presentations and the rarity of the disease. However, these malignant splenic vascular neoplasms can be misdiagnosed as benign vascular tumors or other nonvascular tumors due to the high variability in histology [2]. We present a 56-year-old female with a history of symptomatic anemia and thrombocytopenia with massive splenomegaly secondary to primary splenic angiosarcoma.

## 2. Case Report

A 56-year-old female presented to the hospital with the complaint of fatigue, chest pain, shortness of breath, and dyspnea on exertion. On further review of symptoms she admitted to diffuse left upper quadrant tenderness and unintentional weight loss of 15 pounds. Her past medical history was significant for anemia, hypothyroidism, and a 40-pack-year history of smoking. The patient was suffering from longstanding symptomatic normocytic anemia and was followed up by gastroenterology for a potential etiology. However she underwent several endoscopies and colonoscopies with unsuccessful attempts to diagnose her anemia. The patient received multiple packed red blood cell transfusion therapies for symptomatic relief.

**Figure 1:**
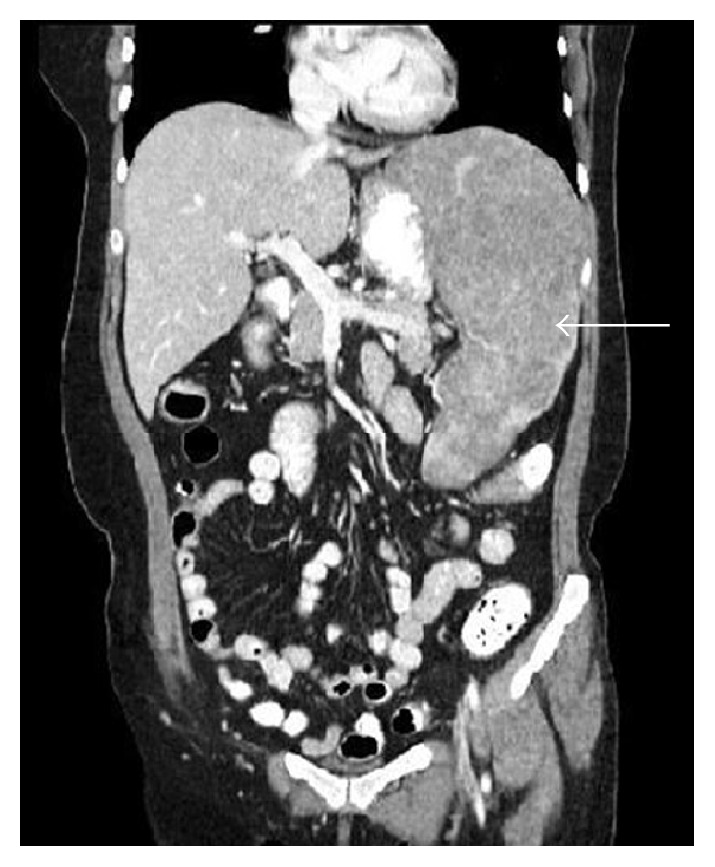
CT scan shows marked enlargement of the spleen and heterogeneous composition and demonstrates low attenuation masses.

**Figure 2:**
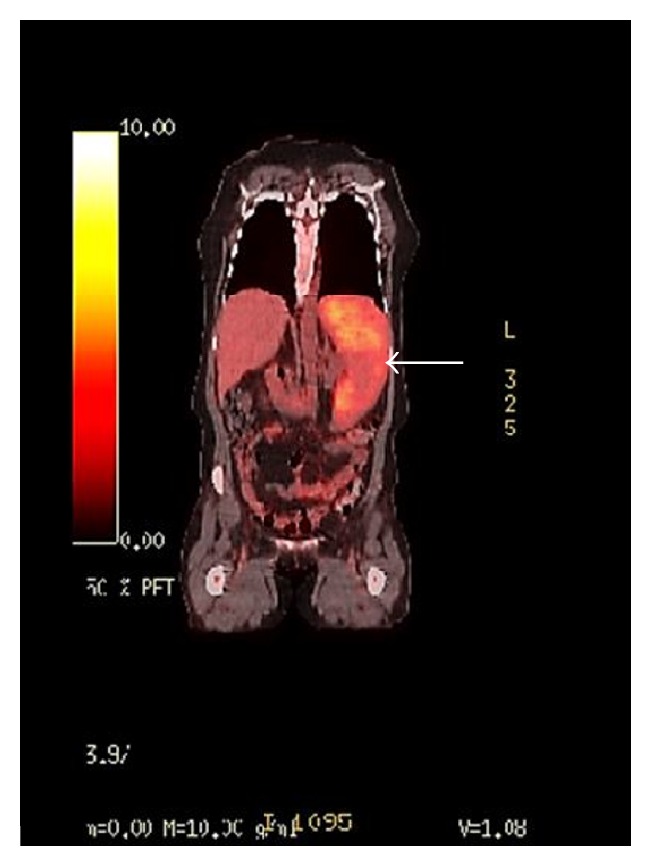
PET scan demonstrating an enlarged spleen with hypermetabolism.

Pertinent laboratory findings included white blood cell count of 15.3 × 10^9^/L, hemoglobin count of 7.1 g/dL, hematocrit of 23.4%, platelet count of 153 × 10^9^/L, MCV of 83.4, and red cell distribution width of 19.7%. A computed tomography with contrast of the abdomen and pelvis demonstrated marked splenomegaly that is heterogeneous with multiple low attenuation masses (Figure 1). In addition the patient underwent a positron emission tomography scan which showed splenic hypermetabolism (Figure 2).

**Figure 3:**
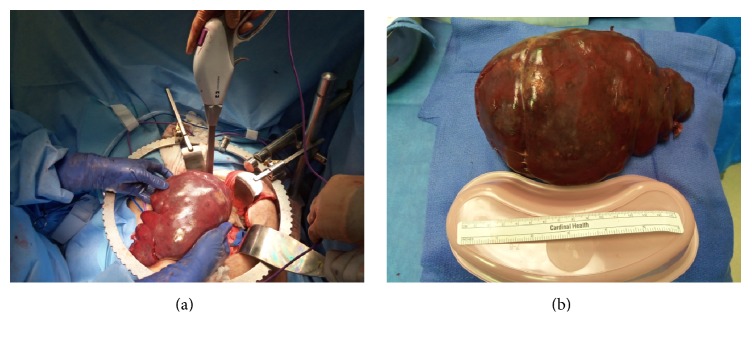
(a) Splenectomy with distal pancreatectomy and (b) enlarged dark brown, soft, and rubbery spleen measuring 19.0 × 13.0 × 10.5 cm.

With no resolution of her symptomology and radiographical evidence of an enlarged hypermetabolic spleen, the patient decided to undergo an elective splenectomy. Prior to the operation interventional radiology performed a splenic artery embolization to reduce blood flow to the spleen. The patient was taken to the operating room for a laparotomy. Upon entering the abdominal cavity the spleen was easily identified and had multiple omental adhesions. Due to the size, increased vascularity, and proximity to surrounding organs the splenic vein completely adhered to the distal pancreas and was unable to be safely dissected away. This resulted in a distal pancreatectomy. Once the spleen was removed the total weight measured 1,124 grams (Figure 3).

**Figure 4:**
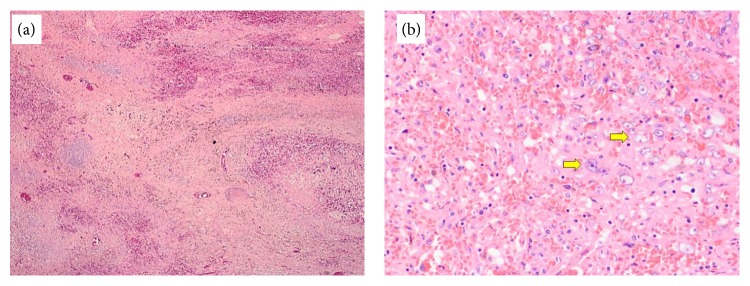
(a) Low power magnification (4x), showing effaced splenic architecture with large areas of hemorrhage. (b) Higher power magnification (20x) showing numerous large atypical cells (arrows).

**Figure 5:**
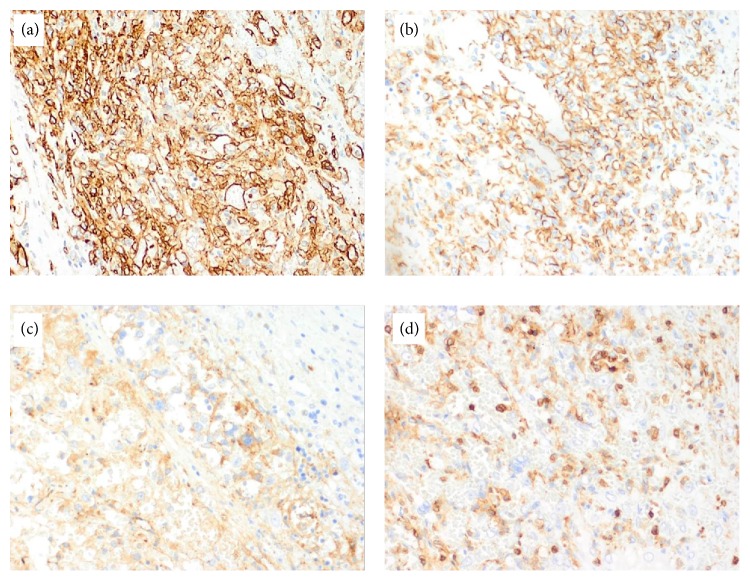
Immunohistochemistry shows the large tumor cells are positive for vascular markers (a) CD34, (b) CD31, and (c) vWF. Only small residual lymphocytes are positive for (d) CD45, and the large atypical cells are negative.

The organ was sent for pathological analysis, with routine sections showing sheets and clusters of large atypical cells with vesicular chromatin and prominent nucleoli in a background of extensive necrosis, lymphodepletion, and abundant macrophages (Figure 4). The histologic differential diagnosis for this lesion is broad and includes a large cell lymphoma (B-cell or T-cell), metastatic carcinoma, melanoma, and vascular neoplasms such as angiosarcoma and Kaposi sarcoma. Immunohistochemical staining identified that the large atypical cells were vascular in origin, being positive for vascular markers CD31, CD34, and von Willebrand factor (vWF). Ki-67 showed a proliferation index of 5–10% (Figure 5). The neoplastic cells were negative for numerous hematolymphoid markers: CD3, CD20, CD8, CD45, CD61, CD138, lysozyme, and myeloperoxidase (MPO). Epithelial marker pan-cytokeratin (cytokeratin AE1/AE3), neuroectodermal/melanocytic marker S-100, and a viral stain for human herpes virus 8 (HHV8) were all negative. Following the confirmed diagnosis, the patient decided to seek further management with oncology. The patient underwent chemotherapy regiment of Adriamycin and ifosfamide with mesna for six cycles and tolerated the subsequent treatment. The patient was amendable to starting paclitaxel. Despite a multimodal treatment, follow-up magnetic resonance imaging demonstrated bone metastasis to the vertebral bodies of cervical and thoracic spine and leptomeningeal metastatic disease involving thoracic cord and conus. However the patient has remained alive more than 12 months from her initial diagnosis.

## 3. Discussion

Angiosarcoma is a malignant neoplasm that originates from vascular endothelial cells. These uncommon neoplasms account for approximately 2% of all soft tissue sarcomas with an increase in incidence over the last 30 years [3]. Angiosarcoma most commonly involves the skin and superficial soft tissues. However other organ systems such as breast, liver, spleen, and bone are affected infrequently. Primary angiosarcomas of the spleen are exceedingly rare occurring at an annual incidence rate of 0.14 to 0.25 cases per million [4]. Splenic angiosarcoma is considered the most common primary nonlymphoid and nonhematopoietic malignant tumor of the spleen [1, 5]. Unlike angiosarcomas that arise in other organ systems, there is no known association between splenic angiosarcoma and occupational exposure to chemicals such as vinyl chloride, arsenic, or usage of contrast agent thorium dioxide [4]. While this malignancy can develop at any age it has been shown that often the mean age at diagnosis is fifty-nine with peak incidence in the 6th decade of life with no sexual predilection [2, 6]. In particular this report describes a 56-year-old female patient who had no known exposure for the development of angiosarcoma with a diagnosis of primary angiosarcoma of the spleen.

The gross and microscopic features of primary splenic angiosarcoma vary considerably which often makes the diagnosis a challenge. Gross macroscopic examination following splenectomy often demonstrates enlarged spleens weighing 250 to 3,200 grams with massive splenomegaly defined as mass greater than or equal to 1,000 grams [2]. In our case the spleen weighed 1,124 grams and thus occupied a large portion of the upper abdomen. Malignant splenic vascular tumors may be mistaken for benign vascular or malignant nonvascular tumors due to the variability in histology [2]. This microscopic structure is typically heterogeneous in nature. While low power views typically demonstrate a nodular appearance, high power examination invariably reveals infiltrative tumor cells [7]. While there are several different malignant morphologic subtypes, the vasoformative form is more frequent than the poorly differentiated solid sarcomatous pattern [6]. The vast majority demonstrate a spongiform or “honeycomb-like” proliferation which is comprised of an irregularly anastomosing network of slit or capillary-like spaces containing erythrocytes [7]. Often these spongiform areas can combine with cavernous cystic spaces and resemble cavernous hemangiomas with papillary fronds of proliferating tumor cells protruding into the vascular lumina of the cystic spaces. While these architectural patterns are similar to those seen in other benign vascular neoplasms, what distinguishes angiosarcoma is the cytologic atypia of the lining endothelial cells which provides clear evidence of malignancy [7]. Neuhauser et al. describe additional features that help distinguish from benign vascular tumors such as foci of necrosis, hemophagocytosis, hyaline globules, and extramedullary hematopoiesis [2]. These neoplastic masses are generally composed of malignant spindled, polygonal, epithelioid, primitive round and multinucleated giant tumor cells. The patient described in the report ultimately was diagnosed with splenic angiosarcoma that resembled a vasoformative pattern that contained large areas of cytologic atypia with vesicular chromatin and prominent nucleoli. These malignant atypical cells also produced numerous capillary-like channels. In addition there were notable sections of extensive necrosis, lymphodepletion, and hemophagocytosis.

Histologic examination shows splenic angiosarcoma is a morphologically heterogeneous entity. The prominent epithelioid and malignant mesenchymal features observed in these neoplasms emphasize the need to examine multiple sections of tissue. The most sensitive and specific means of defining the atypical cells as endothelial in origin is through immunohistochemical staining of tissue [2]. However biopsy is often dangerous and contraindicated due to the risk of rupture; therefore staining is performed following splenectomy. The differential diagnosis for splenic angiosarcoma includes other vascular tumors such as hemangiomas, littoral cell angioma, lymphangioma, hemangiopericytoma, and epithelioid vascular tumors [4]. A panel of immunohistochemical studies that include markers of vascular and histiocytic differentiation may be quite helpful in confirming both the vascular origin of the tumor and histiocytic differentiation which is unique to primary splenic angiosarcomas [2]. The vast majority of these neoplasms demonstrate reactivity for at least two markers of vascular differentiation. Markers for vascular differentiation include CD31, CD34, VEGF3, or von Willebrand factor (vWF). According to Kutok and Fletcher, CD31 is the most sensitive and specific marker usually demonstrating reactivity in even the most poorly differentiated tumors which furthers the diagnosis splenic angiosarcoma [7]. The marker CD34 further distinguishes atypical cell origin with splenic sinusoidal cells staining negative and endothelial cells of small vessels resulting in a positive stain [2]. In addition, these tumors often are positive for at least one marker for histiocytic differentiation such as CD68 and/or lysozyme [7]. Occasional reactivity to Ki-67 and S-100 protein has been reported in several cases showing increased cellular proliferation [2]. The positive staining for the aforementioned markers and the absence of other antigenic markers such as CD3, CD5, CD61, CD138, MPO, PANCK, and HHV8 is essential for confirming diagnosis. Tissue analysis for our patient showed the tumor cells stained positive for CD31, CD34, CD68, S-100, vWF, and Ki-67. This pattern of staining along with the microscopic tissue analysis confirmed the diagnosis of primary splenic angiosarcoma of the vasoformative type. Although long term survivors are rare, no distinguishing histologic feature seems to predict more favorable outcome among patients [7].

Diagnosis is a difficult challenge due to the presence of nonspecific symptoms and the rarity of the disease. This often creates a large differential diagnosis that may not confirm splenic angiosarcoma until a more advanced stage. The spectrum of this disease is highly variable being similar in some aspects to vascular tumors such as hemangiomas, epithelioid hemangioendotheliomas, littoral cell angioma, and Kaposi's sarcoma or malignant nonvascular tumors like lymphangiomas, lymphomas, sarcomas, and secondary metastatic carcinomas [8]. Imaging studies aid in identifying features that narrow the diagnosis. On sonography it is seen as multiple complex heterogenous masses of the spleen [1]. Splenomegaly is the most common imaging finding; however it is present in multiple etiologies. Contrast-enhanced computed tomography of angiosarcomas is usually seen as multiple hypervascular masses in the spleen [1]. These lesions appear as focal round or irregular areas of heterogeneous low attenuation reflecting cystic or necrotic zones. However, lymphoma and metastatic disease frequently affect the spleen and can mimic relatively avascular angiosarcoma [4]. The CT appearance of splenic lymphoma includes homogenous enhancement without a discrete mass, solitary mass, multifocal lesions, and diffuse infiltration, and these masses generally do not enhance or have lower attenuation than the surrounding parenchyma [4]. Splenic lymphoma is often accompanied with enlarged abdominal lymph nodes whereas abdominal lymph node metastasis is less common in angiosarcoma. Although metastatic splenic lesions may mimic a relatively avascular angiosarcoma, there is often evidence of other metastatic lesions associated with the primary neoplasm [4]. In addition, positron emission tomography scan can reliably discriminate between benign and malignant solid splenic masses and illustrate areas of hypermetabolism [9].

Clinically, Falk et al. describe that the most common presenting symptom was abdominal pain that is localized to the left upper quadrant [6]. Often constitutional symptoms of body weight loss, fatigue, and fever accompany abdominal pain. Multiple reports have demonstrated laboratory abnormalities such as anemia, cytopenia, thrombocytopenia, and pancytopenia [6, 10, 11]. The most common type of anemia that accompanies splenic angiosarcoma is normocytic normochromic followed by microcytic normochromic anemia [12, 13]. However when thrombocytopenia is part of clinical history the differential diagnosis expands to include various causes for immune destruction of platelets, hypersplenism, and impaired thrombocyte production [14]. With the increase in mass and vascularity of the spleen, splenic rupture is the most serious manifestation. Rupture may occur in 13% to 32% of cases and at times be the only presenting sign. This also instantly puts patients at risk of peritoneal dissemination with direct implantation of neoplastic tissue or vascular access and hematogenous spread or leads to fatal hemorrhage [15]. This is established since early splenectomy prior to rupture often results in more favorable outcomes. Fortunately the patient presented in this report did not suffer the consequence of splenic rupture. However the clinical history of the patient was significant for longstanding normochromic normocytic anemia, occasional thrombocytopenia, weight loss, fatigue, and left upper quadrant pain.

The prognosis of splenic angiosarcoma continues to remain poor. The median survival of patients diagnosed with angiosarcoma is five to six months without treatment [16]. This survival rate progressively worsens when there are concurrent malignancies. Multiple studies have reported no conclusive means to grade angiosarcoma for prediction of survival [2, 6]. However, Naka et al. reported that tumor size, mitotic counts, and mode of treatment are independent prognostic factors [17]. Metastasis is very common and generally occurs early with the most common site being the liver followed by lungs, lymph nodes, and bone in decreasing frequency [18]. The medial interval between metastasis and recurrence after initial therapy and patient death is 6.5 months [19]. In 9-month follow-up, the patient was found to have metastasis to the vertebral bodies of cervical and thoracic spine and leptomeningeal metastatic disease involving thoracic cord and conus. Death is often secondary to disseminated tumor or, if not treated, splenic rupture resulting in hypovolemic shock and disseminated intravascular coagulopathy [2, 18]. According to the literature the most definitive means of treatment is splenectomy. Several studies have attempted to determine the role and efficacy of chemotherapeutic agents and their effect on outcomes. The study performed by Neuhauser et al. with 28 patients treated with splenectomy involved a minority of patients that received splenectomy with adjuvant radiation, chemotherapy, or combination therapy. However this study resulted in a 93% mortality rate with two patients alive at the final follow-up, one with disease who received combination therapy and the other treated with splenectomy alone who was disease-free [2]. This study also demonstrated that irrespective of treatment, 25 patients died with disseminated disease within 29 months of initial diagnosis with median survival of 5 months. Hsu et al. reported longest disease-free survival following splenectomy at 162 months; however this may have contributed to their more favorable median survival of 36 months following treatment [19]. Despite the differencing in the survival rates, splenic angiosarcoma remains a highly aggressive and lethal neoplastic disease.

Primary angiosarcomas arising from the viscera are extraordinarily rare and there is no current chemotherapeutic standard. In the management of soft tissue sarcomas, doxorubicin and ifosfamide as single drugs were able to demonstrate response rates of 16–36% and are the most active agents [20]. A large meta-analysis of 2,185-patient follow-up data using the anthracycline-based chemotherapy in soft tissue sarcomas found an overall response rate of 26% and median survival of 51 weeks [21]. A historical review of more than 30 years of treatment for advanced soft tissue sarcomas has led to the use of ifosfamide and doxorubicin as first-line treatment in younger patients with good performance status [22]. However these combination regimens, usually containing anthracyclines, have response rates up to 35–60% but have increased toxicity compared with single agents [20]. The multivariate analysis of 55 cases in Japan revealed that the multimodal treatment, wide local excision or amputation followed by chemotherapy with or without radiotherapy, was associated with a more favorable prognosis [17]. While the meta-analysis of adjuvant doxorubicin based chemotherapy improved the time to local and distant recurrence, it was unable to provide evidence for overall survival benefit [23]. While a number of cytotoxic agents have been used in treatment of angiosarcoma, the main agents stem from the treatment of sarcomas with the use of anthracyclines, ifosfamide, and taxanes [3]. Combination therapy of ifosfamide and doxorubicin was used by Cunningham et al. to treat a case of primary ovarian angiosarcoma, yet the patient expired within 7 months of initial diagnosis [24]. Prospective multicentric phase II clinical trial of weekly paclitaxel for metastatic or locally advanced angiosarcoma showed a median time to progression was 4 months, and overall survival was 7.6 months [25]. Following the elective splenectomy, the patient in the report agreed on adjuvant combination chemotherapy of doxorubicin and ifosfamide with mesna. In addition, the patient will be undergoing a trial of paclitaxel for further treatment. The multimodal approach in the treatment plan for this patient helped the patient survive more than 12 months since her initial diagnosis.

## 4. Conclusion

Primary splenic angiosarcoma is a rare and aggressive neoplasm that has a dismal prognosis. Diagnosis and treatment pose a significant challenge. While angiosarcoma of other organs is often related to occupational exposure to vinyl chloride, arsenic, or usage of contrast agent thorium dioxide, this relationship does not exist for the spleen. Although there are many several symptoms associated with this malignancy, none is specific for splenic angiosarcoma. Angiosarcoma of the spleen should be suspected in patients with continuous left upper quadrant pain, splenomegaly, systemic symptoms of malignancy, and persistent hematologic abnormalities. Radiographical imaging is a useful tool in determining the composition of the spleen, size, and metabolism; it alone is not adequate for diagnosis. However definitive diagnosis is made by tissue analysis with immunohistological staining. The current and accepted therapy is splenectomy. While there are some studies using chemotherapy and radiation, there is no specific treatment for splenic angiosarcoma. Early diagnosis and surgical management remain paramount.
